# *Bacillus* Strains Most Closely Related to *Bacillus nealsonii* Are Not Effectively Circumscribed within the Taxonomic Species Definition

**DOI:** 10.1155/2011/673136

**Published:** 2011-10-20

**Authors:** K. Kealy Peak, Kathleen E. Duncan, Vicki A. Luna, Debra S. King, Peter J. McCarthy, Andrew C. Cannons

**Affiliations:** ^1^USF Center for Biological Defense, College of Public Health, University of South Florida, 3602 Spectrum Boulevard, Tampa, FL 33612-9401, USA; ^2^Department of Botany and Microbiology, University of Oklahoma, 770 Van Vleet Oval, Norman, OK 73019-6131, USA; ^3^Harbor Branch Oceanographic Institute at Florida Atlantic University, 5600 US No. 1 North, Fort Pierce, FL 34946, USA

## Abstract

*Bacillus* strains with >99.7% 16S rRNA gene sequence similarity were characterized with DNA:DNA hybridization, cellular fatty acid (CFA) analysis, and testing of 100 phenotypic traits. When paired with the most closely related type strain, percent DNA:DNA similarities (% *S*) for six *Bacillus* strains were all far below the recommended 70% threshold value for species circumscription with *Bacillus nealsonii*. An apparent genomic group of four *Bacillus* strain pairings with 94%–70% *S* was contradicted by the failure of the strains to cluster in CFA- and phenotype-based dendrograms as well as by their differentiation with 9–13 species level discriminators such as nitrate reduction, temperature range, and acid production from carbohydrates. The novel *Bacillus* strains were monophyletic and very closely related based on 16S rRNA gene sequence. Coherent genomic groups were not however supported by similarly organized phenotypic clusters. Therefore, the strains were not effectively circumscribed within the taxonomic species definition.

## 1. Introduction

CBD 118 was one of the two first *Bacillus* strains not related to the *B. cereus* group reported to harbor the capsule genes carried on pXO2 by *Bacillus anthracis* (USF Center for Biological Defense (CBD)) [1, 2]. Luna et al. isolated and sequenced the capsule operon (*cap*A*, cap*B*, cap*C*, cap*D, and promoter), *rep*A, *cap*R, *acp*A, *IS*1627, ORF43, ORF48, and ORF61 on a large plasmid in CBD 118 [1]. Its status as a carrier of *B. anthracis* capsule genes spurred research into determining its closest relatives, to aid in circumscribing the reservoir of genes essential for virulence in *B. anthracis*. When near full length 16S rRNA gene sequences were compared, the most similar type strains to strain CBD 118 were *Bacillus circulans* ATCC 4513^T^ (98.9%) and *Bacillus nealsonii* DSM 15077^T^ (99.3%). Strain CBD 118 differed from *B. circulans* ATCC 4513^T^ and *B. nealsonii* DSM 15077^T^ for 10 and 12 of 100 phenotypic traits evaluated, respectively. The percentages of DNA:DNA binding in two pairings each of strain CBD 118 to *B. circulans* ATCC 4513^T^ and *B. nealsonii* DSM 15077^T^ were 12.5 and 10.2% and 10.8 and 8.3%, respectively. Thus, strain CBD 118 is differentiated by phenotypic and genome-based methods from the only validly named species with greater than 98.7% 16S rRNA gene sequence similarity [3–5]. Strain CBD 118 was the sole exemplar of a novel species. Prior to the proposal of novel species, studies of ten or more strains are recommended in order to detail intraspecies diversity and to foster appropriate type strain assignment [6–8]. To identify the requisite closely related strains, the V1–V3 hypervariable regions of the 16S rRNA gene [9] from strain CBD 118 were compared to sequences available in GenBank. Eight potential sibling strains were obtained for study. Although the eight strains tested negative for capsule production and for the pXO2 genetic marker by PCR, the group retained taxonomic—if not biodefense—significance. This work presents the polyphasic taxonomic characterization of these eight strains with respect to CBD 118. Incongruent strain-strain associations within this polyphasic data set illustrate the difficulties in applying a pragmatic, taxonomic, bacterial species definition to groups of strains that do not fall into coherent clusters based on genetic and phenotypic analyses. 

Bacterial species are currently defined by pragmatic criteria in a coordinated, polyphasic scheme of 16S rRNA sequence-based phylogeny, indirect whole genome comparisons by DNA:DNA hybridization and analysis of numerous covariant phenotypic characters [3, 5, 10, 11]. Key requisites of the taxonomic species definition can be condensed as follow: (i) a species should be a monophyletic group with a high degree of genetic similarity, (ii) the recommended thresholds of 70% DNA similarity and 5°C Δ*T*_*m*_ are guidelines, not absolute limits for circumscribing new species, (iii) genomic boundaries for a separate species should be defined after analysis of the collective phenotype, (iv) phenotypic intragroup homo- or heterogeneity can only be understood after analysis of as many traits as possible among at least five and preferably more strains, (v) a bacterial species should not be classified unless it can be recognized by multiple independent methods and possesses a set of determinative phenotypic properties [3, 5, 11].

Underlying these guidelines are assumptions about the genetic and phenotypic characteristics of bacterial species that may not be equally applicable to all groups of bacteria [12–16]. That is, it is usually assumed that there are clusters of strains, for example, “sequence clusters” [17], “ecotypes” [18], and so forth, distinct from other clusters. Investigators have been encouraged to develop other genomic-based methods to supplement or even supplant DNA:DNA hybridization as the acknowledged standard for delineating genospecies clusters [3, 4, 6, 16, 19]. Various methods are increasingly used to define genetic and phenotypic similarity among strains—from multilocus sequence typing (MLST) [20] up to the analysis of whole genomes [13, 14]. Ever more precise and detailed descriptions of similarity among strains and between clusters can be obtained by advances in sequencing technology, its application to more isolates and by polyphasic phenotypic analysis of increased numbers of characters. But a more fundamental and less tractable problem is that of the species level circumscription of related bacteria that do not appear to fit readily into sequence clusters and hence within the current taxonomic species definition [14]. Taxonomic species definitions continue to be refined as new techniques become available and new strains are described [3, 4, 6, 16, 19]. Our study illustrates complexities that can be encountered as polyphasic methods are applied to greater numbers of strains forming a broader sample of the microbial world. 

## 2. Materials and Methods

### 2.1. Bacterial Strains

Nine *Bacillus* strains in this study were deposited in the Agricultural Research Service Culture Collection (ARSCC), U.S. Department of Agriculture, Peoria, Ill, USA. Accession numbers for the CBD collection and the ARSCC (NRRL) follow the original strain identifiers. *Bacillus* sp. CBD 118 = NRRL B-51264 was isolated from a powder initially suspected of harbouring *B. anthracis* [1]. *Bacillus *strains provided by colleagues: OSS 25 = CBD 1278 = NRRL B-59473 [21]; P307 = CBD 1284 = NRRL B-59474 and P308 = CBD 1285 = NRRL B-59475 (Harbor Branch Marine Microbial Culture Collection); C4T1F3B3 = CBD 1286 = NRRL B-59476 [22]; IAFILS6 = CBD 1287 = NRRL B-59477 [23]; AD5A = CBD 1288 = NRRL B-59478, U4A = CBD 1289 = NRRL B-59479 and ADP4II = CBD 1290 = NRRL B-59480 [24]. Nucleotide sequence data reported are available in the DDBJ/EMBL/GenBank databases under accession numbers DQ374636, EU656111, EU683686, FJ554672, FJ943256–FJ943261. The type strains of *B. circulans,* ATCC 4513^T^ and of *B. nealsonii*, DSM 15077^T^ were acquired from the American Type Culture Collection (ATCC) and the Deutsche Sammlung von Microoganismen und Zellkulturen (GmbH) (DSMZ), respectively.

### 2.2. Preservation and Authentication of Bacillus Strains

Upon receipt, each strain was subcultured by streaking to tryptic soy agar (TSA) and TSA with 5% sheep red blood cells (TSA-BA) and grown at 30°C. After 24, 48, and 72 h incubation, plated strains were examined for purity based on the presence of colonies of a single morphotype. A single, well-isolated and representative colony was designated as the progenitor colony and streaked for pure culture reisolation on plates of TSA, TSA-BA, and TSA with 5 mg L^−1^ MnSO_4_, incubated at 30°C for up to 72 h. Characteristic, well-isolated colonies on these plates served as first passage sources of inocula for initial phenotypic characterization as detailed below. Colony morphologies for each strain were observed at 24 and 48 h for consistency with the progenitor colony and were described for standard colony features including color, surface texture and degree of luster, relative transmittance of direct light through the colony, shape, margin configuration, elevation, diameter in mm and hemolysis reaction. Each of the *Bacillus* strains in this study including the type strains presented one or more differential colony features that were documented and subsequently monitored as evidence of purity and authenticity whenever strains were subcultured. Phenotypic tests and other procedures utilizing broths were routinely subcultured at the incubation end point to TSA-BA check plates. After 24 and 48 h incubation at 30°C, check plates were reviewed for the presence of colonies of a single, differential morphotype, characteristic of each strain. 

The *Bacillus* including type strains were inoculated from the progenitor colony to aerated tryptic soy broth (TSB), grown to late log phase, subcultured to a TSA check plate, aseptically harvested by centrifugation, resuspended in TSB with 10% glycerol, aliquoted to multiple cryovials, and subjected to a controlled freeze prior to storage at −85°C. One week after cryostocking, a cryovial of each strain was thawed, subcultured on TSA and TSA-BA plates, enumerated for viability and again evaluated for the single, differential colony morphotype. Prior to retesting of phenotypic characters and other analyses, strains were subcultured from the cryostocks and endospore production was induced on TSA with 5 mg L^−1^ MnSO_4_ plates. Serial transfer of strains was restricted by the use of single, characteristic endospore-producing colonies as inoculation sources for subsequent testing. 

Preservation of strain authenticity was evaluated at the end of the study. Four strains that formed an apparent genomic cluster were subcultured from cryopreserved stock, retested for seven differential phenotypic traits including colony morphotype and resequenced for the 16S rRNA gene. The resultant sequences were compared to the original sequences deposited in GenBank.

### 2.3. 16S rRNA Gene Sequence Analysis

Amplification of 16S rRNA gene sequences from *Bacillus* strains, sequencing of the approximately 1500 bp long products, fragment assemblies and alignment of 16S rRNA gene sequences from type strains of selected *Bacillus* species were performed as previously reported [2]; an additional primer, 534R, was employed in some amplifications. Identification of phylogenetic neighbors was carried out by BLAST 2.2.20+ [25] and megaBLAST (discontinuous option) [26] searches of GenBank [27]. Calculation of pairwise sequence similarity to nearest neighbors used the EzTaxon global alignment algorithm [28]. Alignments of 16S rDNA sequences were also made using the Infernal secondary structure based aligner and SeqMatch scores (S_ab) calculated with RDP10 at the Ribosomal Database Project website [29]. Nucleotide (nt) positions in the hypervariable regions V1–V3 were identified in the 16S sequence of strain CBD 118 by alignment with conserved regions at nt positions 48–70, 346–366, and 490–511 in *rrn*E of *B. subtilis* subsp. *subtilis* strain 168^T^ (NC_000964.2, Locus tag: BSUr022, GeneID: 2914197, updated 3/2010 to NC_000964.3, Locus tag: BSU_rRNA_30, GeneID: 8303085) [9]. In the *E. coli* numbering system, regions V1–V3 correspond to nt positions 69–99, 137–242 and 433–497, respectively [30]. Using GenBank bl2seg, pairwise alignments of 461 bp from strain CBD 118 (nt 26–461 corresponding to *rrn*E nt positions 48–511) were made to 16S rDNA sequences from closely related strains. Presumptive signature sequences (PSS) within the V1–V3 region were identified and compared to all GenBank sequences using BLASTN 2.2.20+ with parameters adjusted for short input sequences [25]. Dendrograms were constructed from approximately 1390 bp or 448 bp using neighbor-joining, maximum parsimony and maximum likelihood algorithms (PHYLIP v. 3.6.80) [31] with 1000 bootstrap replications performed to estimate support for each branch.

### 2.4. DNA:DNA Hybridization Studies

Strains were subcultured from cryopreserved stock, grown to late log phase in aerated TSB, harvested by centrifugation and provided to the Deutsche Sammlung von Microoganismen und Zellkulturen (GmbH) (DSMZ) as ≥3 g wet weight biomass preserved in 50:50 sterile dI H_2_O:2-propanol. Prior to harvesting, each broth culture was screened for a single characteristic morphology in wet mounts using phase contrast microscopy at x1000 under oil and subcultured on TSA-BA check plates grown at 30°C. After 24 and 48 h incubation, check plates corresponding to the preserved biomass for each strain were reviewed for the presence of colonies of a single morphotype, consistent with that previously determined to be characteristic of and differential for the strain. Biomass was shipped only after no apparent evidence of contamination or mislabeling of strains was detected. 

DNA:DNA hybridizations were performed by the Identification Service of the DSMZ. Cells of preserved biomass were disrupted in a French pressure cell and the DNA purified by chromatography on hydroxyapatite. DNA:DNA hybridization was carried out at 65°C using a model Cary 100 Bio UV/VIS spectrophotometer equipped with a Peltier-thermostatted 6 × 6 multicell changer and a temperature controller with in situ temperature probe (Varian) [32, 33].

### 2.5. Cellular Fatty Acid Analysis

Cellular fatty acid (CFA) composition of *Bacillus* strains was determined by Microbial ID, Inc. (MIDI) at both study start and end point. Each strain was subcultured from cryopreserved stock, inoculated from a single, characteristic colony to a TSA slant and grown at 30°C for 48 h to foster endospore formation. Prior to shipment, each slant was subcultured on a TSA-BA check plate grown at 30°C and observed after 24 and 48 h incubation. The check plate for each strain was reviewed for colonies of a single, differential morphotype and the slant shipped to MIDI only after no evidence of contamination or mislabeling of strains was discerned.

Strains were grown under standardized conditions on tryptic soy broth agar quadrant streak plates at 28°C for 24 h. To reduce disparities in the effective physiological age of the cells, biomass was harvested from colonies growing in the third streaked quadrant. Fatty acid methyl esters were extracted by a four-step procedure of saponification, methylation, extraction and sample clean-up. Fatty acid peaks were analyzed by gas chromatography and named by comparing retention times to those in a known mixture. A dendrogram program used a multivariate clustering algorithm to produce unweighted pair matching based on similar CFA content between strains and generated a tree scaled to Euclidian distance (ED).

### 2.6. Phenotypic Characterization

All tests were incubated at 30°C unless otherwise noted [34]; incubation periods are specified. Differential tests were performed at minimum twice or as specified; prior to re-testing, strains were subcultured from cryostocks held at −85°C in TSB, 10% glycerol. Control strains of *Bacillus* and *Paenibacillus* included *B. cereus* ATCC 14579^T^, *B. circulans* ATCC 4513^T^, *B. megaterium* ATCC 14581^T^, *B. nealsonii* DSM 15077^T^, *B. pumilus* ATCC 7061^T^, *B. thuringiensis* ATCC 10792^T^, and *P. polymyxa *ATCC 43865^T^. Sporulation was induced on TSA with 5 mg L^−1^ MnSO_4,_ grown for 40–48 h. Hemolysis reaction was determined on TSA with 5% sheep red blood cells (REMEL), grown for 48 h. Pigment production and mean colony diameter were evaluated on TSA, tryptone blood agar base and tryptone glucose yeast extract plates, 24, 48, 72 h and 1 week. Motility was determined by either stab inoculation of motility test medium (REMEL), observed at 24 and 48 h, or phase contrast observation of wet mounts made with aerated cells grown in TSB to log phase, 3 to 6 h. Cell morphology, endospore characterization and swelling of the sporangium, presence of parasporal bodies and motility were observed in wet mounts using phase contrast microscopy at ×1000 under oil. Anaerobic growth was evaluated after 1 week in the Mitsubishi Pack-Anaero anaerobic gas generating system with the following pre-reduced media: fluid thioglycollate medium with dextrose and indicator (REMEL), tryptone glucose yeast extract agar plates, and anaerobic agar [34], inoculated in the molten state. Oxidase reaction was tested with Kovács' phenylenediamine redox dye reagent (Becton, Dickinson). Growth of cells at defined temperatures was tested in 3 mL of TSB in 13 × 100 mm tubes for 48 h in water baths set to 30, 35, 40, 45, 50, 55, and 60 + 1°C and examined for turbidity at 24 h intervals. Growth of cells at defined pH was tested in the same manner in TSB adjusted to pH 4.6, 5.6, 6.1, 6.5, 6.8, 7.3, 7.8, 8.1, and 8.5. Salt tolerance was tested on nutrient agar plates supplemented with 0, 1, 3, 7 and 10% NaCl, incubated for 5 days. Physiological tests performed on cells grown in commercial media (REMEL) included casein and starch hydrolysis, incubated 14 days; growth on mannitol egg yolk polymyxin agar, incubated 48 h; growth in methyl red Voges-Proskauer (MRVP) broth for final pH and VP reaction, tested at 3, 5, and 7 days; nitrate reduction tested in nitrate broth at 3, 7, and 14 days and on nitrate agar slants, at 3 and 5 days; and growth on Sabouraud's 4% glucose agar, pH 5.6, incubated 72 h. Gelatin hydrolysis was tested in 12% nutrient gelatin (REMEL) for 2 weeks. Hydrolysis of Tween-80 was tested on plates of a peptone-based medium [35], incubated for 4 weeks. Acid production from 49 carbohydrates or carbohydrate derivatives was tested using the API 50 CH panel and API CHB/E medium with mineral oil overlay, ≥4 test panels per strain, in combination with eleven biochemical tests from the API 20 E kit, ≥2 test panels per strain, incubated for 48 and 24 h, respectively (bioMérieux). Acid production was read at 24 and 48 h in a semiquantitative way, where 0 was assigned to negative reactions of the same alkaline red as the no-carbohydrate control and 5 assigned to yellow indicator shifts of maximum intensity. Values of 1, 2, 3, or 4 were given to intermediate reactions with 3, 4, and 5 being considered positive. Differential phenotypic traits between paired strains were enumerated. Each differential character state was assigned a numerical value—that is, 1 = negative, 2 = variable, 3 = positive—and subjected to hierarchical cluster analysis (SPSS for Windows, Release 15.0.1.1, 2007). A dendrogram was generated using average linkage between groups, scaled in Euclidian distance units.

## 3. Results and Discussion

### 3.1. Bacterial Strains Obtained for Taxonomic Study

Eight *Bacillus *strains having ≥99.7% 16S rRNA gene sequence similarity [19, 36] and ≥0.980 SeqMatch S_ab scores to 1495 bp of strain CBD 118 (DQ374636) were provided to the CBD for taxonomic comparisons: strain OSS 25 (EU683686), isolated from a metallurgic waste site in Italy [21]; strains P307 (FJ943260) and P308 (FJ554672), isolated from deep-water marine sponge (*Discodermia* sp.), Bahamas; strain C4T1F3B3 (FJ943258), isolated from cultured flounder, USA [22]; strain IAFILS6 (FJ943259) isolated from a consortium degrading polyaromatic hydrocarbons, Canada [23]; strains AD5A (FJ943256), U4A (FJ943261), and ADP4II (FJ943257), isolated from plant thorns, Israel [24]. The most closely related type strains were *Bacillus circulans* ATCC 4513^T^ (AY724690) (98.9%; S_ab not ranked) and *B. nealsonii* DSM 15077^T^ (EU656111) (99.3%; S_ab 0.961).

### 3.2. Presumptive Signature Sequences

Hypervariable regions V1–V3 in the *Bacillus* 16S rRNA gene sequence had been reported to be discriminatory for most *Bacillus* species [9]. The eight *Bacillus* strains were identified as potential sibling strains when 461 bp spanning V1–V3 hypervariable regions of the 16S rRNA gene sequence of strain CBD 118 were compared to GenBank database sequences. Presumptive signature sequences (PSS) were identified in V1 at nt positions 71–92 (PSS_1_A and PSS_1_B) and in V2 at nt positions 183–223 (PSS_2_) (Table 1). The eight *Bacillus* strains, strain CBD 118 and *B. nealsonii* DSM 15077^T^ differed from *B. circulans* ATCC 4513^T^ by two nucleotide changes in PSS_1_ and at five positions in PSS_2_. *B. nealsonii* DSM 15077^T^ also differed in PSS_2_ at four nucleotide positions and by insertion of two thymines. Since not all 16S rRNA sequences from *B. circulans* and *B. nealsonii* strains have been examined for these signatures, the PSS are termed presumptive. Strains CBD 118 and OSS 25 were identical for 461 bp spanning V1–V3 and differed from the other seven *Bacillus* strains by one bp in PSS_2_a (Table 1). In nucleotide blast searches of the GenBank database, eight sequences—including that of strain CBD 118—had 100% coverage and 100% identity to both PSS_1_B and PSS_2_a. When 1495 bp from *Bacillus* sp. CBD 118 (DQ374636) was used as the reference sequence, four near full length sequences from cultured strains (EU683686, *Bacillus* sp. OSS 25; DQ333291, *Bacillus benzoevorans* LLG; EU660368, *Bacillus nealsonii* CT18; GU471201, *Bacillus* sp. Q2CJ3) each had ≥99.8% similarity, suggesting all four were *Bacillus* strains more closely related to CBD 118 than to *B. benzoevorans* or *B. nealsonii *type strains. Searches coupling PSS_1_B with PSS_2_b or PSS_2_c identified other isolates with >99% sequence similarity to strains C4T1F3B3, P307 and P308 and to IAFILS6, AD5A, U4A, and ADP4II, respectively.

### 3.3. Phylogenetic Analysis

**Figure 1:**
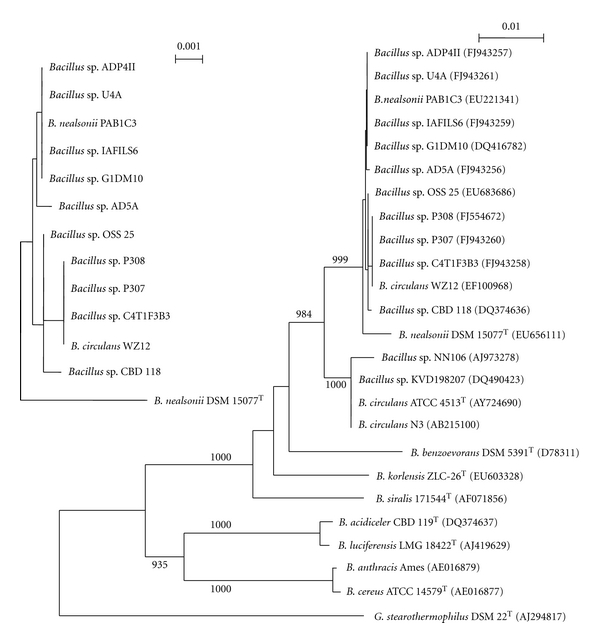
Phylogenetic relationship of strain CBD 118 with type strains of other *Bacillus* spp. and with strains from GenBank with highest similarities (accession numbers in parentheses). The Neighbor-Joining tree and subtree are based on approximately 1390 bp of the 16S rRNA gene. Numbers show the level of bootstrap support from 1000 repetitions with only those >750 shown. Bars, nucleotide substitutions per nucleotide.

In a neighbor-joining (N-J) tree (Figure 1) based on approximately 1390 bp of 16S rRNA sequence, strains most closely related to *B. circulans *and* B. nealsonii* were divided into two well-supported sister clades. Strain CBD 118 and *Bacillus* strains with ≥99.7% sequence similarity to CBD 118 formed a complex clade with *B. nealsonii* DSM 15077^T^. In the subtree (Figure 1), the *Bacillus* strains grouped according to PSS_2_ type, but without strong bootstrap support. The *B. circulans* clade included *B. circulans* ATCC 4513^T^, one strain identified as *B. circulans *and two* Bacillus *spp. with ≥99.5% similarity to the type strain. The attribution of species-level identity based solely on 16S rRNA gene sequence similarity is known to be unreliable [6, 7] especially among *Bacillus* [36]. Keswani and Whitman [36] studied the relationship of 16S rRNA sequence similarity (*S*) to DNA:DNA hybridization (*D*). Among 40 *Bacillus* spp., for an *S* of 0.997, 0.993 or 0.991, *D* could be expected to be <0.70 about 50, 95 or 99% of the time, respectively. Strains OSS 25 and WZ12 in the *B. nealsonii *clade were identified in GenBank as *B. circulans*, however each had <99% sequence similarity to *B. circulans* ATCC 4513^T^, indicating the strains do not belong to that species [36]. *Bacillus benzoevorans* DSM 5391^T^ (D78311) was placed outside the *B. circulans* cluster. Strains C4T1F3B3, AD5A and U4A in the *B. nealsonii *clade were identified in GenBank as *B*. *benzoevorans;* however, each had 96% sequence similarity to *B. benzoevorans* DSM 5391^T^, significantly below the threshold for species level relatedness [19, 36]. Sequences of strains OSS 25, C4T1F3B3, AD5A and U4A, when compared to the EzTaxon database of curated type strain sequences, were most closely related to *B. nealsonii *DSM 15077^T^ (99.3%, 99.3%, 99.4%, and 99.5%, resp.). Phylogenies of N-J, maximum parsimony, and maximum likelihood trees [31] (not shown) were consistent in placement of strains into the same clades described above, whether based on 1390 bp or 448 bp encompassing the hypervariable regions. Based on 16S rRNA gene sequence-based phylogeny, the strains gathered for this study were considered to be *Bacillus* spp., most closely related to *B. nealsonii.*

### 3.4. % DNA:DNA Similarity (% S)

DNA:DNA pairings were conducted between *B. nealsonii* DSM 15077^T^ and two strains representing each PSS_2_ type—CBD 118, OSS 25 (PSS_2_a); P308, C4T1F3B3 (PSS_2_b); IAFILS6, AD5A (PSS_2_c) (Table 2). DNA:DNA% similarity (% *S*) data reveals clusters of closely-related strains termed genomic groups or genomic species and remains the “gold standard” for species circumscription [3, 5, 19, 37, 38]. More distantly related groups are separated by discontinuities, commonly in the range of 50–70% *S; *usually only a few intermediate strains are found [37, 38]. The % *S* for each of the six *Bacillus *strains to *B. nealsonii* DSM 15077^T^ was less than 40%, well below the recommended 70% threshold value to be circumscribed in that species [3, 5]. The % *S* between PSS_2_a strains CBD 118 and OSS 25 were 49.9% and 55.5%. In a previous set of pairings, % *S* of 63.8 and 61.6 suggest differences in DNA quality between the two testing events. The %* S* for the four CBD 118 and OSS 25 pairings fall within the 70–50% transitional range for species circumscription [6, 7, 37]. Pairings of P308 (PSS_2_b), C4T1F3B3 (PSS_2_b) and IAFILS6 (PSS_2_c) with CBD 118 (PSS_2_a) resulted in at least one % *S* per strain at or very close to the 70% threshold for species delineation with CBD 118.

**Figure 2:**
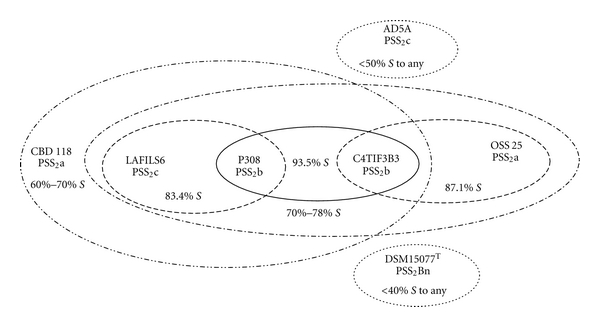
Diagram of averaged % DNA:DNA similarities (% *S*) between *Bacillus* strains DSM 15077^T^, CBD 118, OSS 25, P308, C4T1F3B3, IAFILS6, and AD5A with reference to the presumptive signature sequence 2 type (PSS_2_).

Given the estimated 10% reproducibility of % *S* values (DSMZ), DNA:DNA pairings that have ≥80% *S* should meet or exceed the recommended 70% threshold to delineate taxa at the species level. Three of 21 strain pairings—P308 with C4T1F3B3; OSS 25 with C4T1F3B3; P308 with IAFILS6—tested at ≥80% *S*; therefore, these four strains appeared to represent a coherent genomic cluster. A diagram (Figure 2) in which the two measurements per pairing were averaged, illustrates varying degrees of genomic coherency among all 7 strains. Averaged % *S* of 93.5% strongly supports DNA relatedness between strains P308 (PSS_2_b) and C4T1F3B3 (PSS_2_b); % *S* of 83.4% also supports relatedness between P308 (PSS_2_b) and IAFILS6 (PSS_2_c). But relatedness between C4T1F3B3 (PSS_2_b) and IAFILS6 (PSS_2_c) is not similarly well supported at 70% *S*, thus the degree of relatedness of each strain to P308 was not reproduced in relation to each other. Also, within this apparent genomic cluster, averaged % *S* was 87.1% between OSS 25 (PSS_2_a) and C4T1F3B3 (PSS_2_b), 78.2% between OSS 25 (PSS_2_a) and P308 (PSS_2_b), but was 70.25% between OSS 25 (PSS_2_a) and IAFILS6 (PSS_2_c). A genomic cluster based on these four strains incorporates an ~24% range for % *S* and values for two pairings that may—given ~10% reproducibility—lie in the transitional range for species circumscription. Some strains of a species may show less than 70% *S *with the type strain or other strains of the same species, thus internal heterogeneity within genomic groupings and species is permitted [6, 7, 11, 16]. However, studies of the average nucleotide identity (ANI) of all conserved genes between any two genomes [13, 14, 39] support adoption of a higher rather than a relaxed threshold for species circumscription. The 70% threshold for species delineation based on DNA:DNA pairings corresponds to 95% ANI and 85% or 79% conserved protein coding genes between a pair of strains [39], thus substantial phenotypic differences were possible among two or more of these four strains.

### 3.5. Cellular Fatty Acid Analysis (CFA)

Cellular fatty acid compositions of the nine *Bacillus* strains, *B. circulans* ATCC 4513^T^ and *B. nealsonii* DSM 15077^T^ are compared in Table 3. Consistent with *Bacillus *[34], the major cellular fatty acids (CFA) measured in the strains were C_14:0,_ C_15:0_-anteiso_, _C_15:0_-iso and C_16:0_. Profiles from a second CFA analysis performed at the end of study (not shown) were consistent with those in Table 3. The second data set deviated in the absence of 1–7 very low % CFAs (most <0.5%; three <1.5%) from the profiles of each of the strains, suggesting differences between the two testing events in the effective physiological age of the strains. The ability to reproduce profiles for a single strain is dependent on standardized conditions for growth medium, incubation time and temperature, and effective physiological age of the cells ([34], MIDI technical literature). In the second analysis, slight changes in values of major and other CFAs for ≥9 strains followed a parallel pattern of elevation (C_15:0_-iso, C_17:0_-iso) or reduction (C_14:0, _C_16:1_ *ω*11*c*, C_16:0_), also suggesting differences in effective physiological age for those strains relative to the first testing event. However, salient differential CFAs were reproduced in the profiles of both data sets, supporting the authenticity of the strain set at both time points in our study. C_15:0_-anteiso was 60% in both profiles for strain OSS 25. Strain P307 was twice distinguished by the summed feature C_17:1_-anteiso B/Iso I and C_19:0_-anteiso. In both data sets, PSS_2_c strains IAFILS6, AD5A, U4A, and ADP4II were differentiated from all other strains by C_17:1_ iso *ω*10*c*, the summed feature C_17:1_ anteiso B/Iso I and C_19:0_ anteiso. 

**Figure 3:**
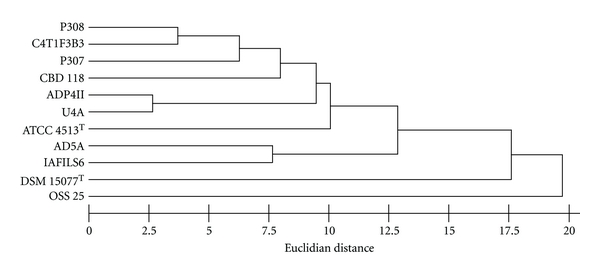
Dendrogram based on cluster analysis of cellular fatty acid composition for 11 *Bacillus* strains including *B. circulans* ATCC 4513^T^ and *B. nealsonii* DSM 15077^T^, in Euclidian Distance units.

CFA profiles are known to vary widely in many named *Bacillus* spp., thus circumscribing species based on CFA content is usually possible only in cases of genomically-homogeneous strains [34]. Therefore, we considered only the linkage of nearest neighbors without attribution of taxonomic level. In a dendrogram scaled to Euclidian distance (ED) and based on the initial data set (Figure 3), the three strains sharing PSS_2_b—P307, P308, and C4T1F3B3—clustered together at near 6 ED. Strain OSS 25 (PSS_2_a) was distantly linked at near 20 ED to the other *Bacillus* spp. including CBD118 (PSS_2_a). Among PSS_2_c strains, ADP4II linked to U4A at ≤3 ED and IAFILS6 linked to AD5A at 7.5 ED but the linkage between the two pairs was at ≤13 ED. In the dendrogram (not shown) based on the end of study data set, small cumulative differences in individual fatty acid percentages relative those in the initial data set resulted in changes in the level of ED linkage among strains. However, the profile similarities between strains P308 and C4T1F3B3, the 60% of C_15:0_-anteiso that distinguished strain OSS 25, and the differentiation of IAFILS6 by C_17:1 _iso *ω*10*c*, summed feature C_17:1_ anteiso B/Iso I and C_19:0_ anteiso were all reproduced in both data sets. In both dendrograms, strains P308 and C4T1F3B3 were clustered at ≤6 ED, IAFILS6 clustered with AD5A at ≥7.5 ED, and OSS 25 was isolated at ≥16 ED. The genome-based cluster of four strains—P308, C4T1F3B3, OSS 25, and IAFILS6—with DNA:DNA pairings of 94–70% *S* was not reproduced in the CFA-based dendrogram from either data set.

### 3.6. Differential Phenotypic Characterization

**Figure 4:**
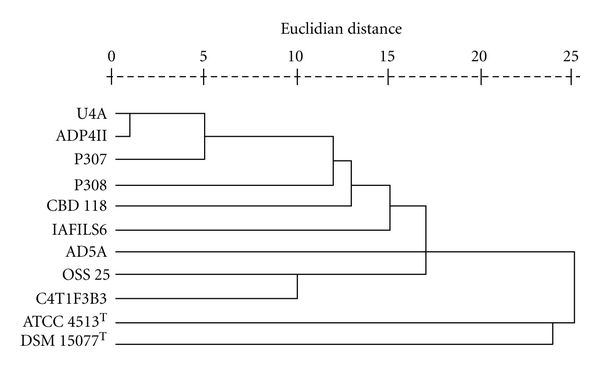
Dendrogram based on cluster analysis of differential phenotypic characters among 11 *Bacillus* strains including *B. circulans* ATCC 4513^T^ and *B. nealsonii* DSM 15077^T^, in Euclidian Distance units.

Twenty-five of 100 phenotypic traits differentiate among the nine *Bacillus *and the two most closely related type strains (Table 4). Type strains *B. circulans* ATCC 4513^T^ and *B. nealsonii* DSM 15077^T^ are distinguished from the other strains by lack of acetoin production and by acid from 2-ketogluconate. The numbers of characters that separate each pair of *Bacillus* strains were compiled in a matrix (Table 5) and presented in a dendrogram (Figure 4). The only consistency between the CFA-based (Figure 3) and phenotype-based dendrogram was the close linkage of two PSS_2_c strains, U4A and ADP4II—one of only two instances in which strains of the same PSS_2_ type were directly linked in the phenotype-based dendrogram. Only 3 of 36 phenotypic pairings resulted in ≤5 character differences while 19 strain pairs had  ≥10 differences (highest number = 13). As a group, strains IAFILS6, AD5A, U4A and ADP4II sharing PSS_2_c formed the most coherent group with between 3–8 character differences. But in the phenotype dendrogram, closely paired strains U4A and ADP4II clustered with P307 (PSS_2_b), while AD5A links with OSS 25 (PSS_2_a) and C4T1F3B3 (PSS_2_b). Strains U4A and ADP4II differed by one CFA (Table 3) and 3 phenotypic characters, and may represent strain variants of a novel species. Only 6 or 5 characters differentiated P307 (PSS_2_b) from U4A and ADP4II, respectively. DNA : DNA pairing data on these three strains is not, however, available for comparison. The four strains—P308, C4T1F3B3, OSS 25, and IAFILS6—that comprised the genomic-based cluster were differentiated by 9–13 characters, including nitrate reduction, temperature range, and acid production from carbohydrates.

### 3.7. Incongruence of Character Sets and Application of a Bacterial Species Definition

The taxonomic species definition mandates that a species be a monophyletic group with a high degree of genomic similarity that also shares a high order of similarity in many independent phenotypic features [3, 5, 10, 11]. The eight strains collected for comparison to strain CBD 118 are monophyletic (Figure 1) when considering 16S rRNA gene similarity. While recognizing that 16S rRNA sequence lacks resolving power at the level of bacterial species [6, 7, 11, 36], we hypothesized that the PSS types might yet function as exclusionary thresholds, for example, species that shared a PSS_2_ type might or might not be the same species, but strains with different PSS_2_ types would not be the same species. While the highest degree of DNA relatedness (93.5% *S*) was between PSS_2_b strains P308 and C4T1F3B3 (Figure 2), the hypothesis of an exclusionary threshold was contradicted by  ≥70% *S* between strains of different PSS_2_ types. However, we suggest that the PSS_2_ types remain effective tools to search 16S rRNA sequence databases for more strains of >99.7% similarity.

No strain tested at greater than 70%–50% *S* in pairings to CBD 118 (Table 2) and the three strains most closely-related to CBD 118 (PSS_2_a) based on % *S*—P308 (PSS_2_b), C4T1F3B3 (PSS_2_b), and IAFILS6 (PSS_2_c)—can be differentiated by 8, 11 and 12 characters, respectively (Table 5). Strain OSS 25, most closely related to CBD 118 based on 16S rRNA sequence similarity and PSS_2_a, differs by 9 phenotypic characters as well as having only transitional range % *S* to CBD 118 and distant linkage based on CFA. It is recommended for *Bacillus* and related genera [6] that the 70% *S* threshold for species delineation not stand alone in delimiting species but should be supported by other characteristics that differentiate strains of the proposed species from other species. In the application of the taxonomic species definition, phenotype continues to have a salient role in the determination of break-points in genomic data for species circumscription and no single parameter—genomic properties or phenotypic traits—should be given undue prominence [3, 6, 37]. The classification that results from application of the taxonomic species definition should be predictive, establishing determinative properties and therefore cannot be based only on genomic characters [3, 7, 11]. The 70% threshold could be interpreted flexibly [6, 7, 11, 16, 37] and a more relaxed boundary used to circumscribe a genomic grouping of these four strains with CBD 118. The resultant grouping would, however, lack sufficient phenotypic cohesion to be of predictive value and therefore does not justify circumscription as a taxonomic species. 

In polyphasic taxonomic studies when the strains and phenotypic characters tested were both sufficiently numerous, the resultant clustering pattern has generally reproduced the genomic grouping [7]. In this instance, the four strains with highest % *S* to support species circumscription are differentiated by multiple phenotypic, species level discriminators (Tables 2, 3, 5). Strain OSS 25 (PSS_2_a) paired with P308 (PSS_2_b) and with C4T1F3B3 (PSS_2_b) at 78% and 87% *S* (Figure 2), but were differentiated by 11 and 9 characters respectively, as well as significant differences in CFA profiles. In the phenotype dendrogram (Figure 4), OSS 25 was linked most closely with C4T1F3B3 but not with P308. Strains P308 (PSS_2_b) and IAFILS6 (PSS_2_c) share 83% *S* but are differentiated by CFA profiles and 12 traits. Strains P308 (PSS_2_b) and C4T1F3B3 (PSS_2_b) share the strongest DNA relatedness with 94% *S* and were closely linked based on CFA profiles but can be differentiated by 13 phenotypic characters and failure to be linked in the phenotype dendrogram. At the end of the study, these four strains were subcultured from cryopreserved stock, retested for six differential phenotypic traits and resequenced for the 16S rRNA gene. The resultant sequence for each strain was subjected to BLAST analysis and in each case resulted in a 100% match to the region of overlap with the ~1500 bp previously accessioned into GenBank for the strain. For each strain, the re-evaluation of six phenotypic characters—degree of endospore-driven swelling, colony diameter, hemolysis reaction, growth at 45°C, growth with 7% NaCl, and nitrate reduction—reproduced the results of previous testing shown in Table 4. These results indicate that the authenticity of these strains was maintained though the course of the study. The internal diversity of strains P308, C4T1F3B3, OSS 25 and IAFILS6 confounds delineation in a phenotypically coherent unit and their circumscription as one species accommodating multiple biovars or ecovars does not, in our minds, support a predictive taxonomy. No common ecological or disease state can be cited to justify the nomination of a pragmatic species epithet for these strains. The designation of genomovars, as originally proposed by Ursing et al. [38], applies to two or more genomic strain clusters within a phenotypically coherent named species that cannot be phenotypically delimited from other strains of the nomenospecies. With these four strains, the converse is the case—one apparent genomic group of strains with four differential phenotypes. It is possible that each of these four strains is the sole exemplar of a novel species and that cohesive phenotypic clusters await the isolation and robust polyphasic characterization of more sibling strains. On the other hand, MLSA [20] on these and more sibling isolates could support the description of one or more species with a high degree of intraspecies diversity—thereupon, a species description could be justified. At this point, rather than being reinforced by coherent phenotypic clustering, potentially coherent genomic clusters among strains are contradicted by interstrain variability and are not therefore effectively circumscribed within the taxonomic species definition.

Difficulties in applying the taxonomic species definition are not new—see the taxonomic histories of *Pseudomonas stutzeri* [38] and *Acinetobacter *[40] to cite just two—whereas these nine *Bacillus* strains are demonstrably novel and their degrees of relatedness appear to confound the taxonomic species definition. Polyphasic data did not clarify relationships and illuminate coherent clusters among these strains—instead, potentially “transitional” forms were revealed. While acknowledging the current insufficiency of our data set, these strains are reminiscent of Model 9 of Istock et al. [12], “Highly variable partially recombining nonspecies”, in which clusters of strains may be discerned, but transitional strains erase any clear demarcation between clusters. Likewise, these strains may be an example of the “continuum of diversity” suggested to characterize groups in which forces promoting coherence dominate those promoting divergence of populations [13]. More data is required to clarify relationships among these strains—particularly sampling more strains in order to determine the range of variation and whether or not discrete phenotypic clusters exist. Indeed, it is hoped that researchers holding closely related strains recognizable by 100% identity to the PSS_2_ types will join in collaborating with labs having expertise in recommended methods of *Bacillus* identification [6] to characterize an expanded number of strains. To this end, the nine *Bacillus* strains in this study have been deposited in a publicly accessible culture collection.

##  Authors' Contribution

K. K. Peak and K. E. Duncan contributed equally to this work.

## Figures and Tables

**Table 1:** Presumptive signature sequences (PSS) conserved in 16S rRNA gene at nt positions 71–92^a,b^ (PSS_1_) and 183–223^a^ (PSS_2_) in respective V1 and V2 hypervariable regions of *Bacillus nealsonii-*related strains. ^a^Numbering based on *rrn*E of *Bacillus subtilis* subsp *subtilis* strain 168^T^ (NC_000964.3); ^b^gap at nt position 80 in PSS_1_ sequence alignments to *rrn*E is reflected in the numbering.

PSS type	*Bacillus* Strain no.	GenBank Accession no.	Nucleotides of PSS
PSS_1_A	ATCC 4513^T^	AY724690	G A C T T **T** A A A^b^A G C T T G C T T T T **A**
	*B. circulans*		
	N3 *B. circulans *	AB215100	G A C T T **T** A A AA G C T T G C T T T T **A**
PSS_1_B	DSM 15077^T^	EU656111	G A C T T **A** A A AA G C T T G C T T T T **T**
	*B. nealsonii*		
	CBD 118	DQ374636	G A C T T **A** A A AA G C T T G C T T T T **T**
	OSS 25	EU683686	G A C T T **A** A A AA G C T T G C T T T T **T**
	P307	FJ943260	G A C T T **A** A A AA G C T T G C T T T T **T**
	P308	FJ554672	G A C T T **A** A A AA G C T T G C T T T T **T**
	C4T1F3B3	FJ943258	G A C T T **A** A A AA G C T T G C T T T T **T**
	IAFILS6	FJ943259	G A C T T **A** A A AA G C T T G C T T T T **T**
	AD5A	FJ943256	G A C T T **A** A A AA G C T T G C T T T T **T**
	U4A	FJ943261	G A C T T **A** A A AA G C T T G C T T T T **T**
	ADP4II	FJ943257	G A C T T **A** A A AA G C T T G C T T T T **T**
PSS_2_a	CBD 118	DQ374636	CCTTT**T**CTACTCATGTAGGAAAG—CTGAAAGACGGTTTA—CGC
	OSS 25	EU683686	CCTTT**T**CTACTCATGTAGGAAAG—CTGAAAGACGGTTTA—CGC
	CT18	EU660368	CCTTT**T**CTACTCATGTAGGAAAG—CTGAAAGACGGTTTA—CGC
	Q2CJ3	GU471201	CCTTT**T**CTACTCATGTAGGAAAG—CTGAAAGACGGTTTA—CGC
PSS_2_b	P307	FJ943260	CCTTT**C**CTACTCATGTAGGAAAG—CTGAAAGACGGTTTA—CGC
	P308	FJ554672	CCTTT**C**CTACTCATGTAGGAAAG—CTGAAAGACGGTTTA—CGC
	C4T1F3B3	FJ943258	CCTTT**C**CTACTCATGTAGGAAAG—CTGAAAGACGGTTTA—CGC
PSS_2_c	IAFILS6	FJ943259	CCTTTTCTACTCATGTAG**A**AAAG—CTGAAAGACGGTTTA—CGC
	AD5A	FJ943256	CCTTTTCTACTCATGTAG**A**AAAG—CTGAAAGACGGTTTA—CGC
	U4A	FJ943261	CCTTTTCTACTCATGTAG**A**AAAG—CTGAAAGACGGTTTA—CGC
	ADP4II	FJ943257	CCTTTTCTACTCATGTAG**A**AAAG—CTGAAAGACGGTTTA—CGC
PSS_2_ *B. nealsonii *	DSM 15077^T^	EU656111	CCTTTTCTACTCATGTAG**AG**AAG**T**CTGAAAGACGG**CA**T**CT**CGC
PSS_2_ *B. circulans *	ATCC 4513^T^	AY724690	CCTTTTC**CT**CTCATG**AG**G**A**AAAG—CTGAAAGACGGTTTA—CGC

**Table 2:** % DNA:DNA similarity between seven *Bacillus* species including *B. nealsonii* DSM 15077^T^, with reference to presumptive signature sequence 2 (PSS_2_) types. ^a^Duplicate measurements from DNA:DNA hybridizations performed using spectrophotometric determinations.

Strain ID	CBD 118	DSM 15077^T^	OSS 25	P308	C4T1F3B3	IAFILS6	AD5A
PSS_2_ type	PSS_2_a	PSS_2_Bn	PSS_2_a	PSS_2_b	PSS_2_b	PSS_2_c	PSS_2_c
Strain ID							
CBD 118							
DSM 15077^T^	17.8 (11.1)^a^						
OSS 25	49.9 (55.5)	13.9 (19.3)					
P308	63.6 (67.8)	38.6 (29.7)	76.7 (79.7)				
C4T1F3B3	70.8 (66.3)	15.6 (21.8)	85.6 (88.5)	94.0 (93.0)			
IAFILS6	67.9 (68.1)	30.6 (25.4)	72.2 (68.3)	86.2 (80.6)	67.3 (72.7)		
AD5A	49.2 (41.9)	8.1 (6.9)	35.6 (28.2)	19.7 (23.4)	15.6 (25.2)	19.3 (21.1)	

**Table 3:** Comparison of percent cellular fatty acid content between strain CBD 118, eight closely related strains, and most closely related type strains. 1: CBD 118; 2: OSS 25; 3: P307; 4: P308; 5: C4T1F3B3; 6: IAFILS6; 7: AD5A; 8: U4A; 9: ADP4II; 10: *Bacillus nealsonii* DSM 15077^T^; 11: *Bacillus circulans *ATCC 4513^T^. ^†^C17:1 anteiso B/Iso I. Other summed features with composite less than 1% for all strains are not presented. CBD 118 and OSS 25 have PSS_2_a; P307, P308, and C4T1F3B3 have PSS_2_b; IAFILS6, AD5A, U4A, and ADP4II have PSS_2_c.

Strains	1	2	3	4	5	6	7	8	9	10	11
C_13:0_ iso	1.55	1.69	0.91	0.94	1.15	0.72	0.48	0.72	0.76	3.41	0.67
C_13:0_ anteiso	0.24	0.53	0.21	—	0.22	0.24	0.18	—	—	0.79	0.17
C_14:0_ iso	2.52	2.95	1.48	1.73	1.82	1.28	1.81	2.96	1.81	2.39	4.78
C_14:0_	9.93	3.97	6.37	7.90	7.04	4.25	4.71	3.82	3.58	13.67	11.66
C_15:0_ iso	25.95	20.57	20.17	21.53	23.24	16.59	14.68	21.42	21.36	29.28	20.25
C_15:0_ anteiso	42.78	60.69	47.94	43.91	44.73	46.86	51.51	45.18	44.28	35.09	40.64
C_16:1_ *ω*7*c *alcohol	0.23	—	0.20	—	0.21	0.18	0.73	0.60	0.50	0.59	1.02
C_16:0_ iso	1.07	1.32	0.92	1.04	1.08	1.22	1.09	2.54	1.67	0.74	4.36
C_16:1_ *ω*11*c *	2.66	0.33	2.82	2.02	2.23	2.06	4.37	2.69	2.79	4.69	2.64
C_16:0_	8.84	4.22	7.59	12.55	9.58	5.68	3.47	3.73	3.02	4.60	5.58
C_15:0_ iso 3OH	—	0.33	—	—	—	—	—	0.96	1.31	—	—
C_15:0_ 2OH	—	0.61	—	—	—	—	—	0.83	1.42	—	—
C_17:1_ iso *ω*10*c *	—	—	—	—	—	0.28	0.44	0.28	0.26	0.25	0.23
Sum in feature 4^†^	—	—	0.34	—	—	0.50	2.17	1.30	1.20	0.36	0.40
C_17:0_ iso	0.80	0.36	1.22	1.25	1.21	3.09	1.21	1.61	1.54	0.87	1.27
C_17:0_ anteiso	3.41	2.42	9.35	6.90	7.49	16.11	12.15	8.84	10.23	3.27	6.07
C_16:0_ iso 3OH	—	—	—	—	—	—	—	0.42	0.34	—	—
C_16:0_ 2OH	—	—	—	—	—	—	—	0.21	0.54	—	—
C_17:0_ iso 3OH	—	—	—	—	—	—	—	—	0.30	—	—
C_18:0_	—	—	0.26	0.23	—	0.28	0.19	—	—	—	—
C_17:0_ 2OH	—	—	—	—	—	—	0.16	0.59	1.46	—	—
C_19:0_ anteiso	—	—	0.22	—	—	0.66	0.63	0.34	0.66	—	—

**Table 4:** Comparison of differential phenotypic traits for strain CBD 118, eight closely related strains, and most closely related type strains. Strains: 1, *Bacillus circulans* ATCC 4513^T^; 2, *Bacillus nealsonii* DSM 15077^T^; 3, CBD 118; 4, OSS 25; 5, P307; 6, P308; 7, C4T1F3B3; 8, IAFILS6; 9, AD5A; 10, U4A; 11, ADP4II. ^a^Approximate mean diameter of 5 well-isolated colonies grown 24 + 2 h, 30°C on tryptic soy agar plates. ^b^Larger, more opaque zone of hydrolysis for these strains. Subtle, <50% increase in diameter of sporangium for preponderance of cells field^−1^; Overt, ≥50% increase in diameter of sporangium for ≥25% of cells field^−1^; +: positive; −: negative; Scant: optical density just above threshold for unaided detection; v: variable, indicating both + and − reactions for strain in ≥4 tests. Scant and v were differentiated from both + and − reactions in pairwise tabulation of differential traits.

Characteristic	1	2	3	4	5	6	7	8	9	10	11
Swelling of sporangium	Subtle	−	Subtle	Subtle	Overt	Subtle	Overt	Overt	Overt	Overt	Overt
Colony diameter (mm)^a^	2	2	2	0.75	2	2	2	4	1.5	4	3
Hemolysis reaction	−	−	−	alpha	−	−	−	−	−	−	−
Growth at 45°C	+	Scant	Scant	Scant	+	Scant	+	−	−	+	+
50°C	+	−	−	−	−	−	−	−	−	−	−
Growth with 7% NaCl	+	−	+	+	+	−	+	+	+	+	+
Tween-80 hydrolysis	+	−	−	−	Scant	−	−	+	+^b^	+^b^	+^b^
Acetoin production	−	−	+	+	+	+	+	+	+	+	+
Nitrate reduction	−	−	+	−	+	+	−	+	−	+	+
Acid from L-Arabinose	+	+	−	−	−	v	−	−	−	−	−
D-Arabitol	−	v	+	+	+	+	+	+	–	+	+
Dulcitol	−	−	−	−	−	−	+	−	−	−	−
*α*-methyl-D-Glucoside	+	+	+	–	+	+	v	v	+	+	+
Glycerol	+	+	+	−	v	+	−	v	−	−	+
Glycogen	+	−	v	−	v	+	−	−	−	−	−
Inulin	+	−	−	−	−	−	−	−	−	−	−
2-Ketogluconate	+	+	−	−	−	−	−	−	−	−	−
5-Ketogluconate	−	v	−	+	+	+	+	+	−	−	−
D-Lyxose	−	+	−	−	−	−	−	−	−	−	−
*α*-methyl-D-Mannoside	+	v	v	v	−	−	−	−	−	−	−
Sorbitol	+	+	+	+	+	+	−	−	−	+	+
D-Tagatose	−	+	−	−	−	−	−	−	−	−	−
Xylitol	+	−	v	−	−	−	v	−	−	−	−
D-Xylose	+	+	−	−	+	+	−	v	v	v	+
*β*-methyl-D-Xyloside	+	v	−	v	+	+	v	−	+	+	+

**Table 5:** Number of differential phenotypic traits between paired *Bacillus* strains among 100 traits tested with reference to the presumptive signature sequence 2 type (PSS_2_).

Strain ID	CBD 118	OSS 25	P307	P308	C4T1F3B3	IAFILS6	AD5A	U4A	ADP4II
PSS_2_ type	PSS_2_a	PSS_2_a	PSS_2_b	PSS_2_b	PSS_2_b	PSS_2_c	PSS_2_c	PSS_2_c	PSS_2_c
CBD 118									
OSS 25	9								
P307	8	12							
P308	8	11	7						
C4T1F3B3	11	9	10	13					
IAFILS6	12	12	8	12	9				
AD5A	13	12	10	13	10	7			
U4A	10	11	6	10	10	6	5		
ADP4II	9	12	5	8	11	8	7	3	

## References

[1] Luna VA, King DS, Peak KK (2006). *Bacillus anthracis* virulent plasmid pX02 genes found in large plasmids of two other *Bacillus* species. *Journal of Clinical Microbiology*.

[2] Peak KK, Duncan KE, Veguilla W (2007). *Bacillus acidiceler* sp. nov., isolated from a forensic specimen, containing *Bacillus anthracis* pX02 genes. *International Journal of Systematic and Evolutionary Microbiology*.

[3] Stackebrandt E, Frederiksen W, Garrity GM (2002). Report of the *ad hoc* committee for the re-evaluation of the species definition in bacteriology. *International Journal of Systematic and Evolutionary Microbiology*.

[4] Stackebrandt E, Goebel BM (1994). Taxonomic note: a place for DNA-DNA reassociation and 16S rRNA sequence analysis in the present species definition in bacteriology. *International Journal of Systematic Bacteriology*.

[5] Wayne LG, Brenner DJ, Colwell RR (1987). Report of the *ad hoc* committee on the recommendation of approaches to bacterial systematics. *International Journal of Systematic Bacteriology*.

[6] Logan NA, Berge O, Bishop AH (2009). Proposed minimal standards for describing new taxa of aerobic, endospore-forming bacteria. *International Journal of Systematic and Evolutionary Microbiology*.

[7] Rosselló-Móra R, Amann R (2001). The species concept for prokaryotes. *FEMS Microbiology Reviews*.

[8] Tindall BJ, Rosselló-Móra R, Huse B-J, Ludwig W, Kämpfer P (2010). Notes on the characterization of prokaryote strains for taxonomic purposes. *International Journal of Systematic and Evolutionary Microbiology*.

[9] Goto K, Omura T, Hara Y, Sadaie Y (2000). Application of the partial 16S rDNA sequence as an index for rapid identification of species in the genus Bacillus. *Journal of General and Applied Microbiology*.

[10] Buckley M, Roberts RJ (2006). *Reconciling Microbial Systematics & Genomics, a Report Based on a Colloquium, Sponsored by the American Academy of Microbiology*.

[11] Rossello-Mora R, Kämpfer P, Bull AT (2004). Defining microbial diversity—the species concept for prokaryotic and eukaryotic microorganisms. *Microbial Diversity and Bioprospecting*.

[12] Istock CA, Bell JA, Ferguson N, Istock NL (1996). Bacterial species and evolution: theoretical and practical perspectives. *Journal of Industrial Microbiology and Biotechnology*.

[13] Konstantinidis KT, Tiedje JM (2005). Genomic insights that advance the species definition for prokaryotes. *Proceedings of the National Academy of Sciences of the United States of America*.

[14] Konstantinidis KT, Ramette A, Tiedje JM (2006). The bacterial species definition in the genomic era. *Philosophical Transactions of the Royal Society B*.

[15] Maynard Smith J, Smith NH, O’Rourke M, Spratt BG (1993). How clonal are bacteria?. *Proceedings of the National Academy of Sciences of the United States of America*.

[16] Vandamme P, Pot B, Gillis M, De Vos P, Kersters K, Swings J (1996). Polyphasic taxonomy, a consensus approach to bacterial systematics. *Microbiological Reviews*.

[17] Hanage WP, Fraser C, Spratt BG (2006). Sequences, sequence clusters and bacterial species. *Philosophical Transactions of the Royal Society B*.

[18] Cohan FM (2002). What are bacterial species?. *Annual Review of Microbiology*.

[19] Stackebrandt E, Ebers J (2006). Taxonomic parameters revisited: tarnished gold standards. *Microbiology Today*.

[20] Maiden MCJ, Bygraves JA, Feil E (1998). Multilocus sequence typing: a portable approach to the identification of clones within populations of pathogenic microorganisms. *Proceedings of the National Academy of Sciences of the United States of America*.

[21] Sprocati AR, Alisi C, Tasso F, Segre L, Cremisini C, Meddez-Vilas A (2006). Comparison of microbial communities native to three differently polluted ecological niches in the industrial site of Bagnoli (Naples, Italy). *Modern Multidisciplinary Applied Microbiology, Exploiting Microbes and Their Interactions*.

[22] Gauger E, Smolowitz R, Uhlinger K, Casey J, Gómez-Chiarri M (2006). *Vibrio harveyi* and other bacterial pathogens in cultured summer flounder, *Paralichthys dentatus*. *Aquaculture*.

[23] Lafortune I, Juteau P, Déziel E, Lépine F, Beaudet R, Villemur R (2009). Bacterial diversity of a consortium degrading high-molecular-weight polycyclic aromatic hydrocarbons in a two-liquid phase biosystem. *Microbial Ecology*.

[24] Halpern M, Raats D, Lev-Yadun S (2007). Plant biological warfare: thorns inject pathogenic bacteria into herbivores. *Environmental Microbiology*.

[25] Altschul SF, Madden TL, Schäffer AA (1997). Gapped BLAST and PSI-BLAST: a new generation of protein database search programs. *Nucleic Acids Research*.

[26] Zhang Z, Schwartz S, Wagner L, Miller W (2000). A greedy algorithm for aligning DNA sequences. *Journal of Computational Biology*.

[27] Benson DA, Boguski MS, Lipman DJ (1999). GenBank. *Nucleic Acids Research*.

[28] Chun J, Lee JH, Jung Y (2007). EzTaxon: a web-based tool for the identification of prokaryotes based on 16S ribosomal RNA gene sequences. *International Journal of Systematic and Evolutionary Microbiology*.

[29] Cole JR, Wang Q, Cardenas E (2009). The ribosomal database project: improved alignments and new tools for rRNA analysis. *Nucleic Acids Research*.

[30] Chakravorty S, Helb D, Burday M, Connell N, Alland D (2007). A detailed analysis of 16S ribosomal RNA gene segments for the diagnosis of pathogenic bacteria. *Journal of Microbiological Methods*.

[31] Felsenstein J (2008). PHYLIP, phylogeny inference package, version 3.68. http://evolution.gs.washington.edu/phylip.html.

[32] De Ley J, Cattoir H, Reynaerts A (1970). The quantitative measurement of DNA hybridization from renaturation rates. *European Journal of Biochemistry*.

[33] Huss VAR, Festl H, Schleifer KH (1983). Studies on the spectrophotometric determination of DNA hybridization from renaturation rates. *Systematic and Applied Microbiology*.

[34] Logan N, De Vos P (2009). Genus I. *Bacillus* Cohn 1872, 174^AL^. *The Firmicutes, Bergey’s Manual of Systematic Bacteriology*.

[35] Atlas RM, Parks LC (1993). Tween-80 Hydrolysis Medium. *Handbook of Microbiological Media*.

[36] Keswani J, Whitman WB (2001). Relationship of 16S rRNA sequence similarity to DNA hybridization in prokaryotes. *International Journal of Systematic and Evolutionary Microbiology*.

[37] Rosselló-Móra R, Stackebrandt E (2006). DNA-DNA reassociation methods applied to microbial taxonomy and their critical evaluation. *Molecular Identification, Systematics, and Population Structure of Prokaryotes*.

[38] Ursing JB, Rosselló-Móra RA, Garcia-Valdes E, Lalucat J (1995). Taxonomic note: a pragmatic approach to the nomenclature of phenotypically similar genomic groups. *International Journal of Systematic Bacteriology*.

[39] Goris J, Konstantinidis KT, Klappenbach JA, Coenye T, Vandamme P, Tiedje JM (2007). DNA-DNA hybridization values and their relationship to whole-genome sequence similarities. *International Journal of Systematic and Evolutionary Microbiology*.

[40] Dijkshoorn L, Nemec A (2008). The diversity of the genus acinetobacter. *Acinetobacter, Molecular Biology*.

